# Left ventricular deformation and tissue characteristics in hypertrophic cardiomyopathy patients with HFpEF: a CMR study

**DOI:** 10.1186/s12880-025-02110-4

**Published:** 2025-12-08

**Authors:** Jian Liu, Zhengkai Zhao, Qiuyi Cai, Jiangyu Tian, Jin Gao, Hui Liu, Yao Song, Yuheng Huang, Zhuoan Li, Huaibi Huo, Xin Peng

**Affiliations:** 1 Department of Radiology, The Third People’s Hospital of Chengdu, Chengdu, 610000 China; 2 Department of CT, Shaanxi Provincial People’s Hospital, Xi’an, 710000 China; 3 Department of Radiology, The Fourth Affiliated Hospital of Liaoning University of Traditional Chinese Medicine, Shenyang, 110001 China; 4 Department of Radiology, Indiana University School of Medicine, Indianapolis, 46202 USA; 5 Weldon School of Biomedical Engineering, Purdue University, West Lafayette, IN 47907 USA; 6 Department of Radiology, The First hospital of China Medical University, Shenyang, 110001 China

**Keywords:** Hypertrophic cardiomyopathy, Heart failure with preserved ejection fraction, Cardiac magnetic resonance, H_2_FPEF score

## Abstract

**Purpose:**

This study aimed to evaluate left ventricular (LV) deformation and tissue characteristics using cardiac magnetic resonance (CMR) in patients with hypertrophic cardiomyopathy (HCM) and heart failure with preserved ejection fraction (HFpEF), to examine their associations with heart failure status, and to explore the correlations between CMR parameters and the H_2_FPEF score.

**Methods:**

This retrospective study included 105 patients with HCM who underwent 3.0-T CMR. Participants were classified into HFpEF (*n* = 46) and non-HF (*n* = 59) groups according to the 2019 ESC HFA-PEFF algorithm. Global radial strain (GRS), global circumferential strain (GCS), global longitudinal strain (GLS), and corresponding systolic and early-diastolic strain rates were derived using CMR feature tracking. Myocardial tissue characterization included native T1 and T2 mapping, extracellular volume fraction (ECV), and late gadolinium enhancement (LGE). Group differences were assessed with t-tests or chi-square tests. Associations between strain, tissue parameters, and the H_2_FPEF score were evaluated using Spearman correlations. Multivariable logistic regression was performed to identify independent CMR predictors of HFpEF.

**Results:**

Compared with non-HF patients, those with HCM-HFpEF showed significantly reduced LV systolic and early-diastolic strain rates, including sGRSr (*P* = 0.010), sGCSr (*P* = 0.044), sGLSr (*P* = 0.018), and eGLSr (*P* = 0.006). They also demonstrated a higher prevalence and greater extent of LGE, as well as elevated native T1 and ECV values (all *P* < 0.05). Strain parameters correlated significantly with tissue characteristics (native T1 and mean ECV), except for GCS and ECV. In multivariable analysis, drinking, atrial fibrillation, lower LV-eGLSr, and higher ECV in segments with maximal wall thickness were independently associated with HCM-HFpEF. The H₂FPEF score showed weak but significantly correlations with native T1, ECV, and T2 values in both global and hypertrophied myocardial segments (*r* = 0.199–0.252, all *P* < 0.05).

**Conclusions:**

HCM patients with HFpEF exhibit both systolic and diastolic dysfunction, accompanied by increased diffuse and focal fibrosis. Independent predictors of HFpEF include lower LV-eGLSr, higher segmental ECV, atrial fibrillation, and drinking. The H_2_FPEF score shows significant associations with tissue-level abnormalities, highlighting the complementary role of CMR-derived strain and tissue characterization in the early detection and risk stratification of HFpEF in HCM.

**Supplementary Information:**

The online version contains supplementary material available at 10.1186/s12880-025-02110-4.

## Introduction

Hypertrophic cardiomyopathy (HCM) is defined by unexplained left ventricular (LV) hypertrophy and is commonly associated with impaired diastolic function and elevated filling pressures, despite preserved systolic performance [1]. While the widespread adoption of implantable cardioverter-defibrillators, the incidence of sudden cardiac death in HCM has markedly declined, however, heart failure has emerged as a major clinical concern in this population [2]. Nearly half of patients present with heart failure at initial diagnosis, most often in the form of heart failure with preserved ejection fraction (HFpEF), which is associated with more advanced disease and a higher risk of adverse outcomes compared with those without heart failure [3, 4].

HFpEF in HCM patients represents a heterogeneous and multifactorial pathophysiological entity [5]. Emerging evidence suggests that HCM patients with HFpEF exhibit significantly impaired left atrial phasic function compared with those without heart failure [6]. However, LV deformation and myocardial tissue characteristics in this population remain insufficiently defined. Cardiac magnetic resonance (CMR) provides a comprehensive, noninvasive modality to evaluate myocardial structure, function, and tissue composition with high spatial resolution and reproducibility [7]. CMR feature-tracking (CMR-FT) enables quantification of strain and strain rates, which serve as sensitive markers of subclinical systolic and diastolic dysfunction [8]. Reduced LV global early diastolic longitudinal strain rate (eGLSr), global longitudinal strain (GLS), and global circumferential strain (GCS) reflect combined diastolic and systolic impairment in HFpEF [9, 10], yet their utility for identifying HFpEF specifically in HCM remains unclear.

Multiparametric CMR, including native T1 and T2 mapping, extracellular volume fraction (ECV), and late gadolinium enhancement (LGE), allows comprehensive characterization of diffuse interstitial expansion, myocardial edema, and focal fibrosis, thereby providing complementary insights into adverse remodeling processes underlying HFpEF in HCM [11, 12]. Beyond binary classification of HFpEF using ESC diagnostic criteria [13], the H_2_FPEF score offers a semiquantitative, multidimensional measure of HFpEF likelihood. Incorporating this score may offer a more nuanced understanding of the relationship between cardiac remodeling and HF burden in HCM, serveing as a tool for risk stratification rather than diagnosis [1].

Accordingly, this study aimed to comprehensively assess LV deformation and myocardial tissue characteristics using CMR in HCM patients with and without HFpEF, to identify independent CMR predictors of HFpEF, and to examine correlations between imaging metrics and the H₂FPEF score.

## Materials and methods

### Study population

This retrospective study was approved by the institutional review board of Chengdu Third People’s Hospital (approval number: 2025-S-124) and complied with the Declaration of Helsinki. The requirement for informed consent was waived owing to the retrospective design.

**Fig. 1:**
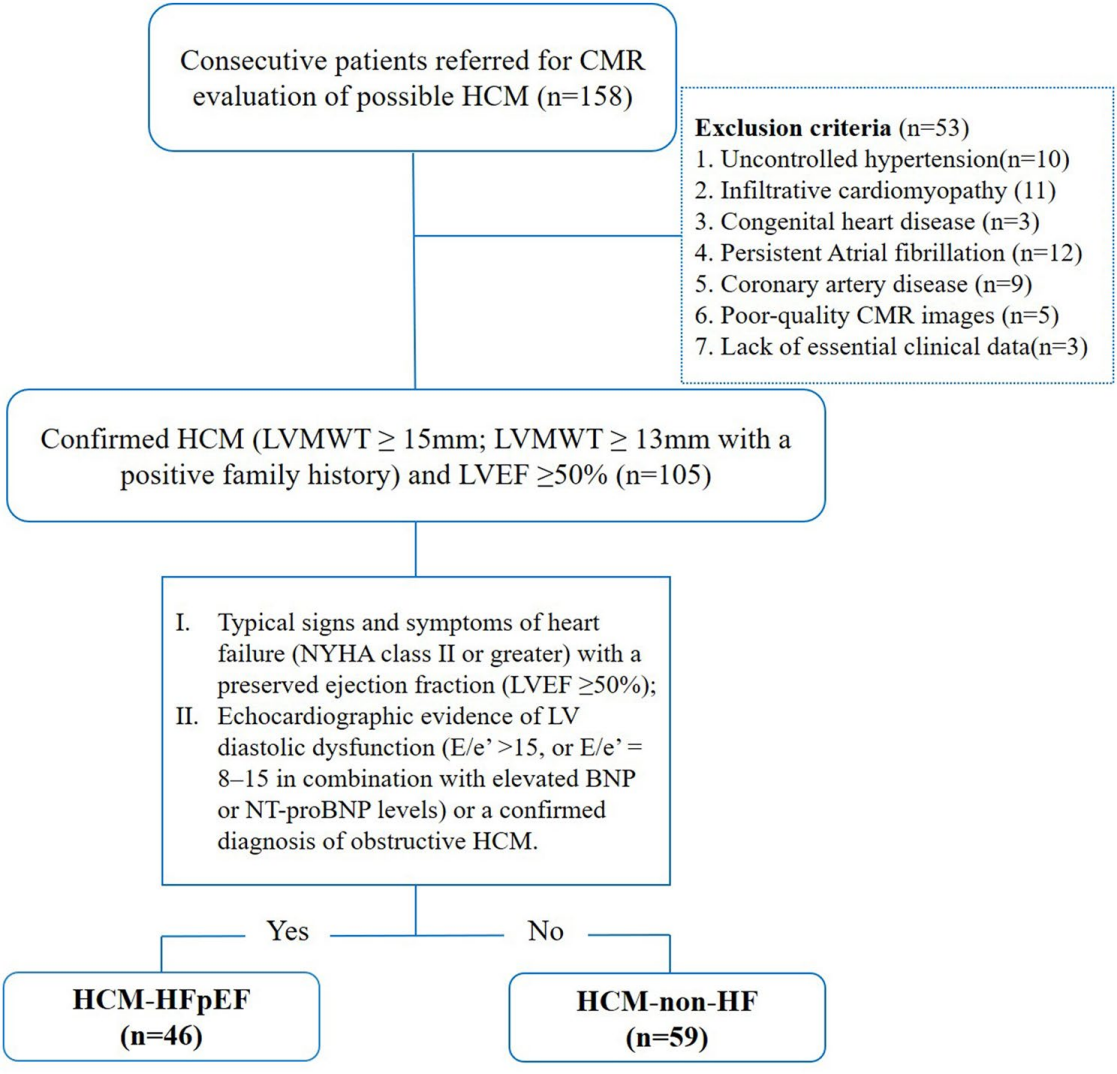
Subject flowchart. CMR, cardiac magnetic resonance; HCM, hypertrophic cardiomyopathy; LVMWT, left ventricular maximal wall thickness; LVEF, left ventricular ejection fraction; HFpEF, heart failure with preserved ejection fraction

A total of 158 patients with a clinical diagnosis of HCM who underwent CMR between June 2020 and July 2024 were consecutively screened. The diagnostic criteria for HCM included non-dilated LV hypertrophy defined by a LV maximum wall thickness (LVMWT) ≥ 15 mm, or LVMWT ≥ 13 mm in the presence of a positive family history, and a preserved ejection fraction (LVEF ≥ 50%), as assessed by CMR [14]. Exclusion criteria included uncontrolled hypertension, infiltrative cardiomyopathies (e.g., amyloidosis), congenital heart disease, significant coronary artery disease (≥ 50% stenosis) or prior myocardial infarction, persistent atrial fibrillation (AF), poor image quality, and missing essential clinical data. Patients with paroxysmal AF were not excluded, provided that they were in sinus rhythm at the time of echocardiography and CMR, as irregular RR intervals during persistent AF may compromise the reliability of diastolic indices (E/e’) and CMR feature-tracking strain analysis. After applying these criteria, 105 patients were eligible for final analysis (Fig. 1).

HFpEF was diagnosed according to the 2019 European Society of Cardiology Heart Failure Association (HFA-PEFF) diagnostic algorithm [6, 15]. Patients were classified into HCM-HFpEF (n = 46) and HCM-non-HF (n = 59) groups. Inclusion criteria for the HCM-HFpEF group included: (I) typical signs and symptoms of heart failure (NYHA class II or greater) with a preserved ejection fraction (LVEF ≥ 50%); and (II) echocardiographic evidence of LV diastolic dysfunction (E/e’ >15, or E/e’ = 8–15 in combination with elevated BNP or NT-proBNP levels) or a confirmed diagnosis of obstructive HCM. Patients who did not meet these criteria were classified as non-HF. The H_2_FPEF score was calculated as a continuous variable to assess HFpEF likelihood, but was not used for diagnostic categorization. Baseline demographic and clinical data were extracted from electronic medical records. Echocardiography (for E/e’ and sPAP measurement) and biomarker assessments (including BNP, hs-cTnT, hematocrit, etc.) were performed within 24 h of the clinical evaluation, and CMR was completed within 48 h of these assessments.

### CMR imaging protocol

CMR examinations were performed on a 3.0T scanner (MAGNETOM Skyra, Siemens Healthcare, Erlangen, Germany). Cine imaging, native T1 mapping, T2 mapping, and LGE sequences were acquired according to standardized protocols. Cine images were acquired using a balanced steady-state free precession (bSSFP) sequence in standard long-axis views (two-, three-, and four-chamber) and contiguous short-axis slices covering the entire left ventricle. Acquisition parameters were as follows: repetition time (TR) = 3.3 ms, echo time (TE) = 1.4 ms, flip angle = 52°, slice thickness = 6 mm. Native T1 mapping was performed using a modified Look-Locker inversion recovery (MOLLI) 5(3)3 acquisition scheme at three short-axis slices (basal, midventricular, and apical levels) with the following parameters: TR = 2.5 ms, TE = 1.1 ms, and flip angle = 35°. T2 mapping was acquired using a T2-prepared single-shot SSFP sequence with three T2 preparation times (0, 25, and 55 ms) at matching short-axis levels. Site-specific reference values for native T1 and T2 at 3.0 T (MOLLI 5(3)3 and T2-prepared SSFP sequence) were derived from our center’s previously published healthy control cohort, which provided validated normal ranges under identical acquisition conditions [16]. LGE imaging was performed 10 min after intravenous administration of gadolinium-based contrast agent (Gadovist; Bayer Healthcare Pharmaceuticals, 0.2 mmol/kg body weight) using a phase-sensitive inversion recovery (PSIR) sequence. Short-axis slices matching those used for cine and mapping sequences, as well as standard long-axis views, were acquired. The inversion time was individually adjusted to null normal myocardium.

### CMR data analysis

Quantitative CMR analysis was independently performed by two experienced radiologists (X.P., 5 years; H.H., 10 years of CMR experience) using Medis Suite software (QMass and QStrain, Medis Medical Imaging Systems, Leiden, the Netherlands). All patients were anonymized and analyzed in a blinded manner.

LV volumes, mass, and LVEF were measured from short-axis cine images. LVMWT was defined as the maximum thickness across all myocardial segments. Myocardial strain analysis was performed using the standard Medis QStrain feature-tracking protocol. GLS was derived from 2-, 3-, and 4-chamber cine images, while GCS and global radial strain (GRS) were obtained from short-axis images. Peak systolic strain rates (sGLSr, sGCSr, sGRSr) and eGLSr were calculated. The software automatically tracked the motion of the endocardium and epicardium to quantify myocardial deformation, with manual adjustments made when necessary to ensure optimal tracking. Papillary muscles and trabeculae were consistently excluded from endocardial border delineation.

**Fig. 2:**
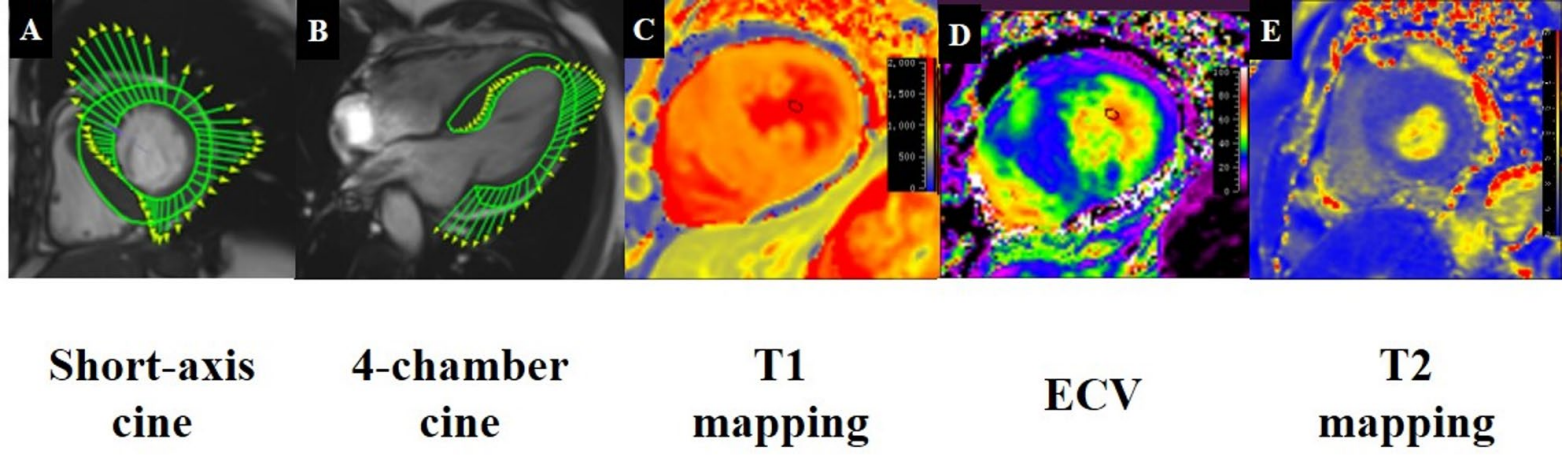
Representative examples of CMR parameter measurements in patients with hypertrophic cardiomyopathy. (**A**, **B**) Strain analysis derived from feature-tracking in one patient. (**C**–**E**) Tissue characterization measurements, including (**C**) T1 mapping, (**D**) ECV, and (**E**) T2 mapping, in another patient. ECV, extracellular volume

Myocardial tissue characterization included T1 mapping, T2 mapping, ECV, and LGE. Global values were obtained from basal, mid, and apical short-axis slices, while regional measurements were performed in hypertrophied segments (segments containing maximal wall thickness, MWT). ECV was calculated using pre- and post-contrast T1 values and hematocrit (Fig. 2). LGE extent was quantified semi-automatically as a percentage of LV myocardial mass using the full-width at half-maximum method, followed by manual correction in QMass.

### Statistical analysis

Continuous variables were expressed as mean ± standard deviation (SD) or median (interquartile range, IQR), and categorical variables as counts (percentages). Normality was assessed using the Shapiro–Wilk test and homogeneity of variance with Levene’s test. Between-group comparisons were performed using the Student’s t-test or Mann–Whitney U test for continuous variables, and the χ² test or Fisher’s exact test for categorical variables, as appropriate. Pearson or Spearman correlation analyses were conducted to evaluate (1) associations between CMR-derived strain parameters and tissue characterization indices, and (2) relationships between the H_2_FPEF score (treated as a continuous variable) and CMR metrics. Correlation strength was interpreted as negligible (< 0.2), weak (0.2–0.39), moderate (0.4–0.59), strong (0.6–0.79), or very strong (≥ 0.8). Univariable logistic regression was first applied to identify potential imaging predictors of HFpEF in HCM. Variables with *P* < 0.10, together with demographic factors (age, sex, body mass index [BMI]) and conventional cardiovascular risk factors (smoking, drinking, hypertension, diabetes, and dyslipidemia), were entered into a multivariable model. To avoid multicollinearity, only one representative was retained from highly correlated variables (e.g., LV-eGLSr over other strain rates; ECV-MWT over global T1 or ECV). The final multivariable model included LV-eGLSr, LGE (%LV mass), ECV-MWT, and the above demographic and risk factors. Odds ratios (ORs) with 95% confidence intervals (CI) were reported. Intra- and inter-observer variability of FT-derived strain and strain-rate measurements were assessed in 20 randomly selected patients. Inter-observer variability was evaluated by two radiologists (X.P. and H.H.) independently analyzing the same datasets, while intra-observer variability was assessed by one radiologist (X.P.) repeating the analysis after a 2-week interval. Agreement was quantified using intraclass correlation coefficients (ICC) and Bland–Altman analysis. All statistical analyses were conducted using SPSS (version 25.0, IBM, Armonk, NY, USA) and GraphPad Prism (version 8.0, GraphPad Software, USA). A two-sided *P* < 0.05 was considered statistically significant.

## Results

### Baseline clinical characteristics

Baseline demographic and clinical characteristics are summarized in Table 1. No significant differences were observed between the HCM-HFpEF and HCM-non-HF groups in age, sex, BMI, heart rate, or the prevalence of conventional cardiovascular risk factors. In contrast, atrial fibrillation and age > 60 years were more frequent in the HFpEF group. Biomarker levels were also significantly higher in HCM-HFpEF patients, with elevated BNP and hs-cTnT compared with non-HF patients. Regarding medical therapy, diuretic use was more common in the HFpEF group, while other treatments were similar between groups.

**Table 1:** Baseline clinical characteristics

Variables		HCM-HFpEF (*N* = 46)	HCM-non-HF (*N* = 59)	*P* value
Age (years)		68 ± 9	59 ± 12	**< 0.001**
Male, n (%)		23(50.0)	31(52.5)	0.796
BMI (kg/m^2^)		25.0 ± 3.9	25.2 ± 3.2	0.833
Heart rate (bpm)		71 ± 13	70 ± 12	0.769
Smoking, n (%)		15(32.6)	25(42.4)	0.307
Drinking, n (%)		15(32.6)	17(28.8)	0.675
Hypertension, n (%)		30(65.2)	32(54.2)	0.256
Diabetes, n (%)		16(34.8)	18(30.5)	0.642
Hyperlipidemia, n (%)		11(23.9)	14(23.7)	0.983
TC (mmol/liter)		4.6 ± 1.9	4.3 ± 1.2	0.285
TG (mmol/liter)		1.9 ± 1.7	2.0 ± 1.5	0.801
LDL-C (mmol/liter)		2.5 ± 0.9	2.5 ± 0.9	0.620
HDL-C (mmol/liter)		1.4 ± 0.3	1.2 ± 0.3	0.103
Hs-cTnT (ng/L)		21.7(12.6–32.1)	13.1(10.0-18.6)	**0.005**
BNP (pg/mL)		244.5(147.4-542.2)	72.3(42.2–108.0)	**< 0.001**
NYHA class, n (%)				
I		0	49(83.1)	
II		44(95.7)	10(16.9)	
III–IV		2(4.3)	0	
H_2_FPEF score variables, n (%)				
H_2_	BMI > 30 (kg/m^2^)	5(10.9)	4(6.8)	0.458
	Arterial hypertension	30(65.2)	32(54.2)	0.256
F	Atrial fibrillation	17(37.0)	5(8.5)	**< 0.001**
P	sPAP > 35 mmHg	9(19.6)	5(8.5)	0.088
E	Age > 60 years	35(76.1)	27(45.8)	**0.002**
F	E/e’ >9	42(91.3)	50(84.7)	0.120
Medical therapy				
Beta-blockers, n (%)		30(65.2)	47(79.7)	0.097
Calcium-channel blockers, n (%)		13(28.2)	13(22.0)	0.463
ACEI/ARB, n (%)		27(58.7)	25(42.4)	0.097
Antithrombotic, n (%)		11(24.0)	11(18.6)	0.510
Diuretic, n (%)		20(43.5)	10(17.0)	**0.003**
Statin, n (%)		30(65.2)	44(74.6)	0.297

Data are reported as mean ± SD, median (IQR), n (%) as appropriate

BMI, Body mass index; TC, Total Cholesterol; TG, Triglycerides; LDL-C, Low-Density Lipoprotein Cholesterol; HDL-C, High-Density Lipoprotein Cholesterol; Hs-cTnT, high-sensitivity cardiac troponin T; BNP, brain natriuretic peptide; NYHA, New York Heart Association; sPAP, systolic pulmonary artery pressure; ACEI/ARB, angiotensin-converting enzyme inhibitor or angiotensin receptor blocker

### CMR-based parameters

Conventional structural and volumetric CMR parameters did not differ significantly between groups (Table 2). Specifically, left ventricular mass (117.1 ± 46.4 vs. 115.4 ± 40.3 g, *P* = 0.835), maximal wall thickness (17.8 ± 4.1 vs. 17.1 ± 3.2 mm, *P* = 0.322), left ventricular mass index (70.7 ± 25.4 vs. 66.6 ± 21.7 g/m², *P* = 0.377), and left ventricular ejection fraction (LVEF, 64.0 ± 7.0% vs. 64.8 ± 5.6%, *P* = 0.504) were comparable between groups. LV volumes (LVEDVi, LVESVi, LVSVi) and cardiac output index (LVCOi) also showed no significant intergroup differences.

**Table 2:** Left ventricular dynamics parameters among subject groups with CMR

Variables	HCM-HFpEF(*N* = 46)	HCM-non-HF(*N* = 59)	*P* value
LVMWT (mm)	17.8 ± 4.1	17.1 ± 3.2	0.322
LVOT obstruction, n (%)	14(30.4)	15(25.4)	0.569
LV mass (g)	117.1 ± 46.4	115.4 ± 40.3	0.835
LV mass index (g/m^2^)	70.7 ± 25.4	66.6 ± 21.7	0.377
LVEDVi (ml/m^2^)	63.5 ± 15.4	64.3 ± 15.3	0.810
LVESVi (ml/m^2^)	23.2 ± 7.9	22.9 ± 7.9	0.860
LVSVi (ml/m^2^)	40.3 ± 10.0	41.5 ± 9.1	0.535
LVEF (%)	64.0 ± 7.0	64.8 ± 5.6	0.504
LVCOi l/(min*m^2^)	2.8 ± 0.9	2.8 ± 0.6	0.483
LV-GRS (%)	50.6 ± 10.4	54.0 ± 9.8	0.089
LV-sGRSr (s^–1^)	1.62 ± 0.36	1.83 ± 0.44	**0.010**
Basal GRS (%)	38.3 ± 13.8	38.6 ± 16.3	0.906
Middle GRS (%)	48.7 ± 17.2	51.8 ± 17.4	0.365
Apical GRS (%)	51.7 ± 24.1	59.3 ± 25.1	0.122
LV-GCS (%)	-23.6 ± 4.7	-23.6 ± 4.4	0.950
LV-sGCSr (s^–1^)	-1.25 ± 0.32	-1.39 ± 0.37	**0.044**
Basal GCS (%)	-23.1 ± 5.6	-23.9 ± 5.6	0.436
Middle GCS (%)	-23.7 ± 5.2	-23.7 ± 5.0	0.970
Apical GCS (%)	-26.5 ± 10.1	-27.5 ± 6.5	0.542
LV-GLS (%)	-20.0 ± 4.2	-21.3 ± 3.7	0.098
LV-sGLSr (s^–1^)	-1.0 ± 0.3	-1.1 ± 0.2	**0.018**
LV-eGLSr (s^–1^)	0.75 ± 0.28	0.90 ± 0.28	**0.006**
Presence of LGE, n (%)	44(95.7)	47(80.0)	**0.017**
LGE (%LV)	6.5(2.2–16.9)	2.9(1.1–11.6)	**0.031**
LV blood T1 (msec)	1789 ± 127	1743 ± 139	0.081
T1 values-mean (ms)	1253 ± 38	1233 ± 28	**0.003**
T1 values-MWT (ms)	1294 ± 53	1264 ± 33	**0.001**
Hematocrit (%)	41.6 ± 5.1	42.9 ± 5.1	0.211
ECV-mean (%)	30.7 ± 2.6	29.7 ± 2.2	**0.030**
ECV-MWT (%)	34.5 ± 4.6	32.4 ± 2.9	**0.005**
T2 values-mean (ms)	40.3 ± 1.7	39.7 ± 1.5	0.068
T2 values-MWT (ms)	40.5 ± 1.7	40.0 ± 1.5	0.093

Data are reported as mean ± SD, median (IQR), n (%) as appropriate

CMR, cardiac magnetic resonance; LV, left ventricular, LVMWT; LV maximal wall thickness; LVOT, LV outflow tract; LVEDVi, LV end-diastolic volume index; LVESVi, LV end-systolic volume index; LVSVi, LV stroke volume index; LVEF, LV ejection fraction; LVCOi, LV cardiac output index; GLS, GCS, and GRS, global peak longitudinal, circumferential, radial strain; sGLSr, sGCSr and sGRSr, global peak systolic LS, CS, RS rate. eGLSr, global early peak diastolic LS rate; LGE, late gadolinium enhancement; MWT, maximal wall thickness; ECV, extracellular matrix volume fraction

### Myocardial deformation and tissue characteristics

**Fig. 3:**
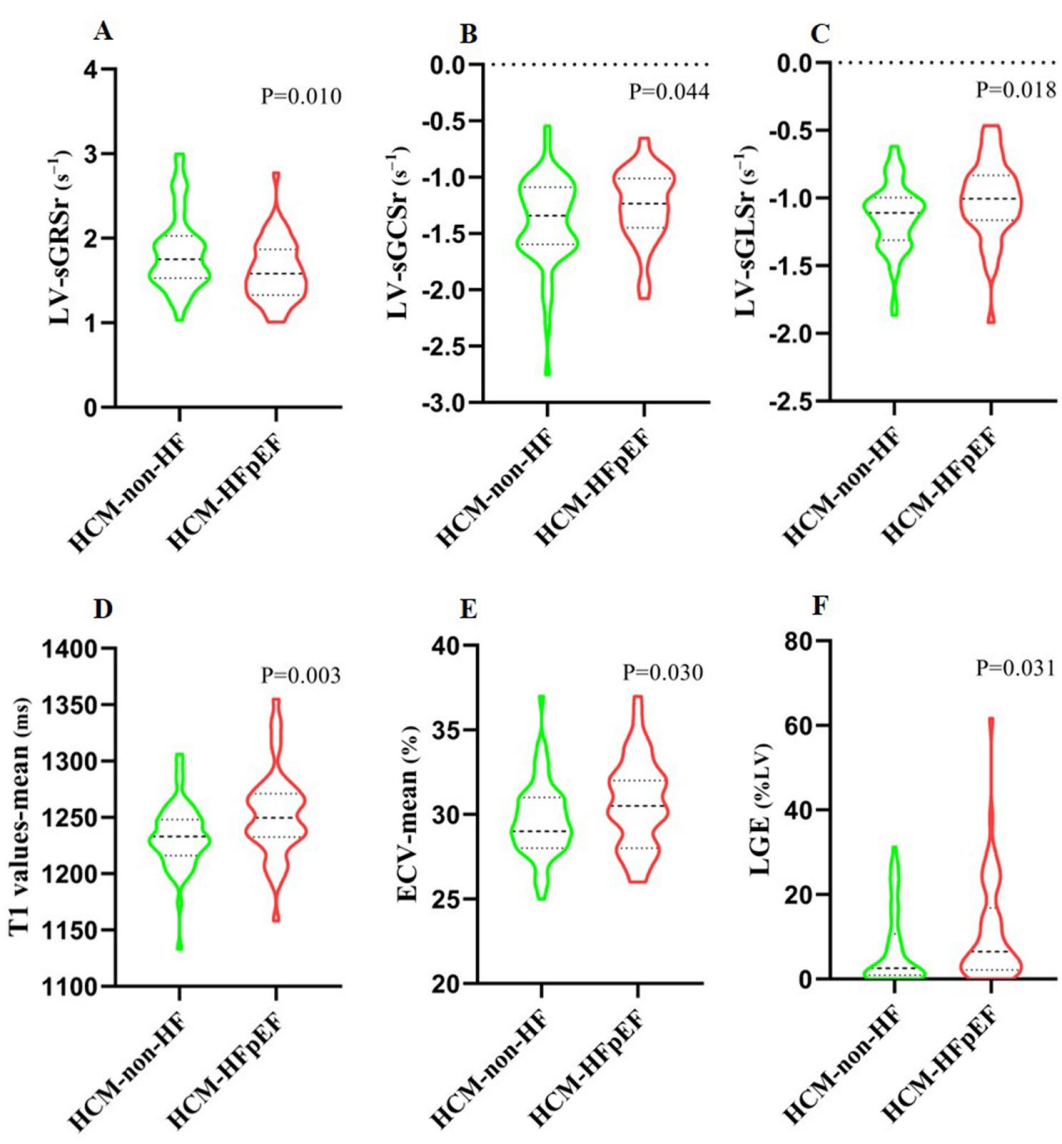
Box-and-whisker plots of CMR parameters in HCM-HFpEF and HCM-non-HF groups. Horizontal lines represent the 5th and 95th percentiles; shaded boxes indicate the interquartile range (25th–75th percentiles); and central lines mark the median values. The plots illustrate group differences in (**A**) LV-sGRSr, (**B**) LV-sGCSr, (**C**) LV-sGLSr, (**D**) mean native T1 values, (**E**) mean ECV, and (**F**) percentage of LGE. sGLSr, sGCSr and sGRSr, global peak systolic LS, CS, RS rate. LGE, late gadolinium enhancement; HFpEF, heart failure with preserved ejection fraction

**Fig. 4:**
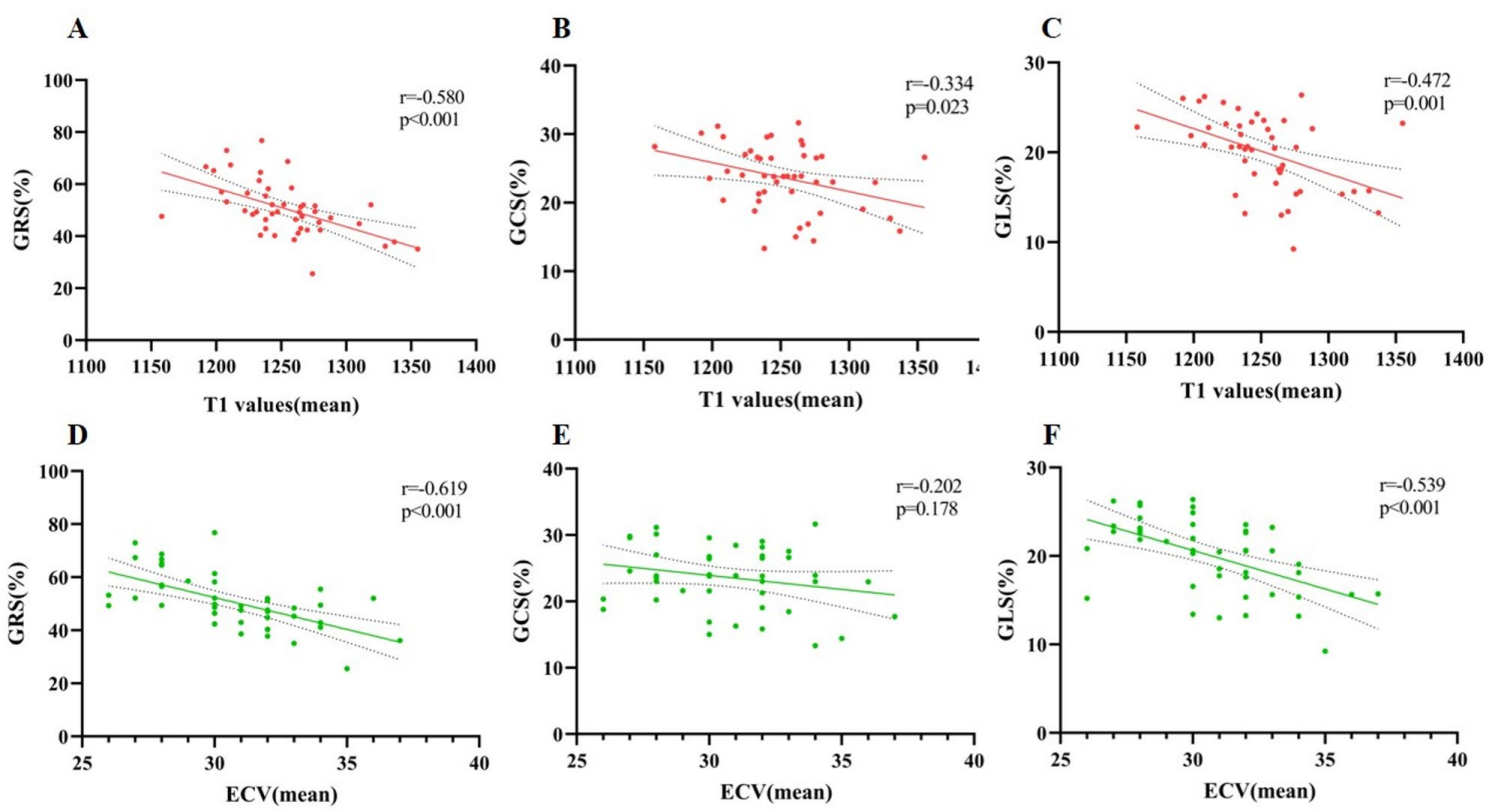
Relationship between CMR-derived myocardial strain and tissue characterization metrics. All parameters showed significant correlations (all *p* < 0.05), except for GCS and ECV (*p* = 0.178)

CMR-derived LV strain parameters and myocardial tissue characterization are summarized in Table 2; Fig. 3. Compared with HCM-non-HF patients, those with HFpEF demonstrated significantly reduced global systolic and early-diastolic strain rates, including sGRSr (*P* = 0.010), sGCSr (*P* = 0.044), sGLSr (*P* = 0.018), and eGLSr (*P* = 0.006). GLS and GRS showed a trend toward lower values in the HFpEF group, but these differences did not reach statistical significance (GLS: *P* = 0.098; GRS: *P* = 0.089), and no significant difference was observed in GCS. Regarding tissue characteristics, HFpEF patients exhibited a markedly higher prevalence of LGE (96% vs. 80%, *P* = 0.017) with greater LGE burden (6.5% vs. 2.9%, *P* = 0.031). Native T1 and ECV values were significantly elevated in the HFpEF group, particularly in segments with maximal wall thickness (T1-MWT: 1294 vs. 1264 ms, *P* = 0.001; ECV-MWT: 34.5% vs. 32.4%, *P* = 0.005). T2 values showed a nonsignificant trend toward higher levels in HFpEF patients. Spearman correlation analysis demonstrated significant associations between CMR-derived strain indices and T1 and ECV (all *P* < 0.05), with the exception of GCS% and ECV, which showed no significant relationship (*P* = 0.178) (Fig. 4).

### Risk factors of HFpEF

Univariable logistic regression identified atrial fibrillation, LV strain rate parameters (sGLSr, sGRSr, sGCSr, eGLSr), and ECV-MWT as potential predictors of HFpEF (all *P* < 0.10). To reduce multicollinearity, LV strain rate indices were first compared, among which eGLSr showed the strongest association with HFpEF, exhibiting the highest odds ratio (Supplementary Table 1, Model 4), and was therefore retained in the final multivariable regression model. After adjustment for demographic and conventional risk factors, four parameters remained independently associated with HFpEF: drinking (OR = 5.79, 95% CI: 1.07–31.33, *P* = 0.041), atrial fibrillation (OR = 7.25, 95% CI: 1.76–29.83, *P* = 0.006), lower eGLSr (OR = 14.97, 95% CI: 2.03–110.60, *P* = 0.008), and higher ECV-MWT (OR = 1.27, 95% CI: 1.05–1.54, *P* = 0.016) (Table 3).

**Table 3:** The association between LV strain and myocardial tissue characterization and HFpEF in HCM patients

Variables	Univariable Analysis		Multivariable Analysis	
	OR (95% CI)	*P* Values	OR (95% CI)	*P* Values
Age per year	1.04(0.99–1.09)	0.078	1.04(0.97–1.11)	0.254
Male sex	0.90(0.42–1.95)	0.796	1.67(0.51–5.40)	0.395
BMI (kg/m2)	0.99(0.88–1.10)	0.831	1.01(0.87–1.17)	0.932
Smoking	1.52(0.68–3.40)	0.308	0.45(0.08–2.35)	0.340
Drinking	0.84(0.36–1.93)	0.675	5.79(1.07–31.33)	0.041
Hypertension	0.63(0.29–1.40)	0.258	1.19(0.42–3.38)	0.740
Diabetes	0.82(0.36–1.87)	0.643	0.31(0.08–1.20)	0.090
Hyperlipidemia	1.01(0.41–2.50)	0.982	0.86(0.24–3.09)	0.816
Atrial fibrillation	6.56(2.19–19.63)	**0.001**	7.25(1.76–29.83)	**0.006**
ECV-MWT (%)	1.18 (1.05–1.33)	**0.006**	1.27(1.05–1.54)	**0.016**
LGE (%LV)	1.04(0.99–1.08)	0.075	1.03(0.98–1.09)	0.242
eGLSr (s^–1^)	9.12(1.75–47.57)	**0.009**	14.97(2.03–110.60)	**0.008**

OR, Odds ratio; CI, confidence interval; BMI, Body mass index; ECV, extracellular matrix volume fraction; MWT, maximal wall thickness; LGE, late gadolinium enhancement; eGLSr, global early peak diastolic longitudinal strain rate

### Association between H_2_FPEF score and CMR parameters

**Fig. 5:**
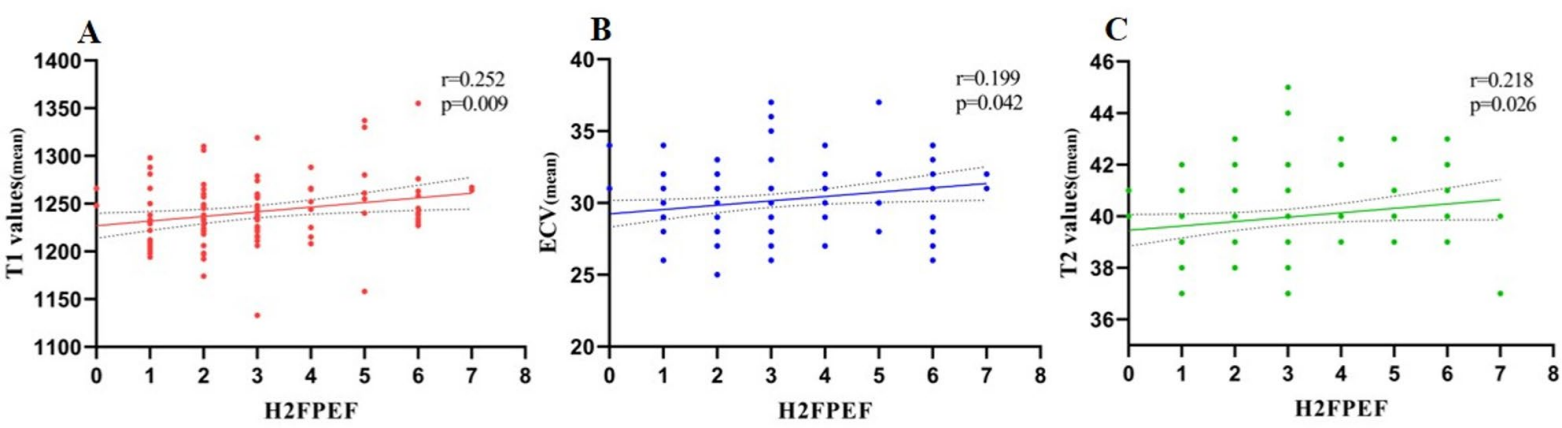
Correlation of T1 values (mean), ECV (mean), T2 values (mean) with H_2_FPEF. Spearman’s correlations were **A** T1 values (mean) and H_2_FPEF: *r* = 0.252, *p* = 0.009; **B** ECV (mean) and H_2_FPEF: *r* = 0.199, *p* = 0.042; **C** T2 values (mean) and H_2_FPEF: *r* = 0.218, *p* = 0.026

Spearman correlation analysis revealed weak to moderate correlations between the H_2_FPEF score and myocardial tissue characterization parameters. Significant correlations were observed with T1-mean (*r* = 0.252, *P* = 0.009), T1-MWT (*r* = 0.249, *P* = 0.010), ECV-mean (*r* = 0.199, *P* = 0.042), ECV-MWT (*r* = 0.240, *P* = 0.014), T2-mean (*r* = 0.218, *P* = 0.026), and T2-MWT (*r* = 0.199, *P* = 0.042), with selected results illustrated in Fig. 5. No significant correlations were observed between H_2_FPEF score and global strain or strain rate parameters.

### Intra and interobserver variability

FT strain and strain-rate measurements demonstrated good intra- and inter-observer reproducibility, with ICCs ranging from 0.81 to 0.99 (Supplementary Table 2). Bland–Altman plots are shown in Supplementary Fig. 1.

## Discussion

This study demonstrates that HCM patients with HFpEF present distinct patterns of myocardial dysfunction and tissue remodeling on CMR. Specifically, compared with HCM patients without HF, those with HFpEF exhibited not only diastolic dysfunction but also impaired systolic function, as evidenced by reduced early-diastolic strain rates (eGLSr) and systolic strain rates (e.g., sGLSr), along with a greater burden of myocardial fibrosis, whereas T2 mapping did not provide additional discriminatory value. Furthermore, drinking, atrial fibrillation, lower LV-eGLSr, and higher ECV in the segment of maximal wall thickness as independent risk factors for HFpEF. In addition, the observed correlations between H_2_FPEF scores and myocardial tissue characterization parameters suggest a potential role for CMR in multiparametric risk stratification of HFpEF among patients with HCM.

Among the deformation indices, GLS(r) reflects longitudinal shortening resulting from endocardial fiber contraction, GCS(r) reflects circumferential shortening resulting from epicardial fiber contraction, and GRS(r) reflects rapid myocardial radial thickening during systole [17]. Strain rate parameters are recognized to be more sensitive than conventional strain indices for detecting early systolic dysfunction and abnormalities in diastolic recoil. Specifically, early-diastolic strain rate serves as a sensitive early marker of diastolic dysfunction [18], whereas peak systolic strain rate reflects regional contractile performance [19]. In HCM patients, subendocardial dysfunction leads to reductions in GLS and GRS, whereas epicardial hypertrophy preserves GCS [20, 21]. In HFpEF, both diastolic impairment, reflected by significantly reduced eGLSr [9, 22], and systolic kinetic abnormalities, indicated by reduced GLS and GCS [10], have been described. Consistent with these prior observations, our findings show that HFpEF in HCM is not purely a diastolic disorder but encompasses measurable systolic kinetic derangements, which are sensitively captured by feature-tracking sGLSr, sGRSr, and sGCS. And HCM-HFpEF patients showed trends toward impaired LV strain (GRS and GLS), although these differences did not reach statistical significance. Notably, LV strain parameters derived from CMR have been shown to correlate with ECV quantified by T1 mapping [23]. In agreement with this framework, our findings highlight pronounced alterations in myocardial tissue composition in HCM-HFpEF. Compared with HCM-non-HF, patients with HFpEF exhibited significantly higher native T1 and ECV values, particularly within hypertrophied segments, along with greater prevalence and burden of LGE, collectively indicating a profibrotic phenotype. This integrated profile of diffuse interstitial expansion (ECV) and focal replacement scarring (LGE) likely contributes to pathological stiffening, impaired relaxation, and functional deterioration characteristic of HFpEF [7, 24–26]. Although the mechanisms of interstitial fibrosis in HCM are incompletely understood, transforming growth factor-β1–mediated extracellular matrix synthesis has been implicated [27]. These findings are consistent with recent evidence indicating that interstitial fibrosis in HCM is closely associated with left atrial remodeling and adverse clinical outcomes, even in patients considered at low risk [28]. Collectively, these observations suggest that diffuse myocardial fibrosis may represent a common substrate linking ventricular diastolic dysfunction, atrial remodeling, and the progression toward heart failure in HCM.

In prior studies, reduced eGLSr has been independently associated with adverse outcomes in HFpEF [9]. In our HCM cohort, impaired longitudinal diastolic deformation—as captured by a lower eGLSr—showed a robust and independent association with HFpEF after adjustment for demographics and conventional risk factors. The independent role of LV-eGLSr underscores the importance of subclinical cardiac dysfunction in HFpEF pathogenesis. Beyond functional markers, ECV-MWT also remained independently associated with HFpEF, with each 1% increment conferring an approximately 30% higher odds of HFpEF. These results are consistent with evidence that alterations in the extracellular compartment are important predictors of adverse outcomes in HCM, including sudden cardiac death, ventricular arrhythmias, and heart failure [27, 29], and that ECV provides prognostic value for MACEs in this population [30, 31]. The coexistence of impaired diastolic strain rate and elevated ECV-MWT supports a mechanistic link between diffuse interstitial expansion and abnormal deformation kinetics. In addition, recent studies have demonstrated that right ventricular (RV) dysfunction carries independent prognostic significance in HCM, beyond left ventricular involvement [32].

The H_2_FPEF score has been shown to be independently associated with HF outcomes in HCM [1]. In our study, no significant correlations were observed with LV strain or strain rate; however, weak but significant associations were identified between native T1, ECV, and T2 values—particularly within hypertrophied segments and in relation to the H_2_FPEF score. This pattern aligns with the understanding that tissue-level alterations precede or occur independently of mechanical changes [33, 34]. Correlation analysis between CMR parameters and the H_2_FPEF score further supports the integrative potential of imaging biomarkers in functional HFpEF assessment. The H_2_FPEF score, while primarily clinical, appears to partially capture myocardial remodeling severity in HCM [35], suggesting that the combination of scoring systems with imaging-derived fibrosis markers could enhance diagnostic and prognostic accuracy. Importantly, the modest correlation coefficients underscore that clinical scores and CMR capture complementary rather than overlapping dimensions of HFpEF pathophysiology.

### Limitations

This study has several limitations. First, it was a retrospective, single-center analysis with a relatively modest sample size, which may have limited the power to detect subtle yet clinically meaningful differences. Second, the retrospective design is inherently prone to selection bias (e.g., overrepresentation of more advanced cases at a tertiary referral center), residual confounding (such as temporal variations in management strategies), and incomplete data on medication adherence, thereby limiting causal inference. Third, CMR was performed at a single time point, precluding assessment of longitudinal changes in myocardial structure and function. Fourth, RV parameters such as RVEF, RV volumes, and RV strain were not available, preventing evaluation of RV remodeling and its potential interaction with the HFpEF phenotype. Finally, several technical limitations inherent to CMR feature tracking should be acknowledged, including inter-vendor variability, limited temporal resolution, dependence on loading conditions [36, 37]. Future studies incorporating comprehensive biventricular assessment, along with larger, prospective multicenter cohorts and serial CMR examinations, are warranted to validate these findings and further delineate the temporal trajectory of myocardial remodeling in HCM-HFpEF.

## Conclusions

In HCM patients, HFpEF is characterized by impaired LV deformation and a profibrotic myocardial substrate. Reduced eGLSr and increased ECV in the segment of maximal wall thickness were independently associated with HFpEF, underscoring the complementary role of functional and tissue-based markers. Weak but significant correlations between the H_2_FPEF score and CMR-derived tissue parameters suggest that integrating clinical scoring with imaging biomarkers may enhance phenotyping and risk stratification. These findings support the utility of multiparametric CMR for earlier detection and individualized management of high-risk HCM patients.

## Supplementary Information

Below is the link to the electronic supplementary material.

Supplementary Material 1

## Data Availability

The datasets used and analysed during the current study are available from the corresponding author on reasonable request.

## Abbreviations

CMR: Cardiac magnetic resonance
GCS: Global circumferential strain
GLS: Global longitudinal strain
GRS: Global radial strain
ECV: Extracellular volume fraction
eGLSr: Global early diastolic longitudinal strain rate
FT: Feature-tracking
HCM: Hypertrophic cardiomyopathy
HFpEF: Heart failure with preserved ejection fraction
LGE: Late gadolinium enhancement
LVEF: Left ventricular ejection fraction
LVMWT: Left ventricular maximum wall thickness
(sGCSr, sGLSr, sGRSr): Global peak systolic CS, LS, RS rate
