# Supplementary material for: Left ventricular deformation and tissue characteristics in hypertrophic cardiomyopathy patients with HFpEF: a CMR study
Source: BMC Med Imaging. 2025 Dec 8;26:26. doi: 10.1186/s12880-025-02110-4 (PMC12801962; doi:10.1186/s12880-025-02110-4)
Supplement: Supplementary file 1 — Supplementary Material 1 [file 12880_2025_2110_MOESM1_ESM.docx]

**Supplementary table 1. Multivariable Associations of LV Strain Rate Subcomponents** **(sGLSr, sGRSr, sGCSr, and eGLSr) With HFpEF in HCM Patients**

| **Variables** | **Modle1** | | **Modle2** | | **Modle3** | | **Modle4** | |
| --- | --- | --- | --- | --- | --- | --- | --- | --- |
|  | OR (95% CI) | P Values | OR (95% CI) | P Values | OR (95% CI) | P Values | OR (95% CI) | P Values |
| Age per year | 1.03(0.96-1.10) | 0.393 | 1.01(0.94-1.01) | 0.810 | 1.02(0.96-1.09) | 0.525 | 1.02(0.96-1.09) | 0.553 |
| Male sex | 1.03(0.32-3.34) | 0.962 | 0.91(0.28-3.00) | 0.879 | 0.98(0.30-3.17) | 0.966 | 1.58(0.46-5.50) | 0.470 |
| BMI (kg/m2) | 1.00(0.87-1.16) | 0.971 | 0.99(0.86-1.12) | 0.908 | 1.02(0.88-1.18) | 0.782 | 1.01(0.87-1.17) | 0.911 |
| Smoking | 0.25(0.04-1.14) | 0.120 | 0.27(0.05-1.49) | 0.132 | 0.21(0.04-1.25) | 0.087 | 0.37(0.06-2.25) | 0.279 |
| Drinking | 5.11(0.87-30.14) | 0.071 | 5.33(0.97-29.18) | 0.054 | 6.09(1.05-35.33) | 0.044 | 6.02(0.96-38.00) | 0.056 |
| Hypertension | 1.35(0.47-3.88) | 0.582 | 1.55(0.55-4.42) | 0.410 | 1.47(0.52-4.17) | 0.467 | 1.29(0.44-3.83) | 0.646 |
| Diabetes | 0.42(0.11-1.57) | 0.198 | 0.34(0.09-1.31) | 0.118 | 0.46(0.12-1.74) | 0.255 | 0.38(0.09-1.52) | 0.171 |
| Hyperlipidemia | 0.90(0.24-3.35) | 0.872 | 1.22(0.33-4.54) | 0.762 | 0.93(0.25-3.43) | 0.915 | 0.90(0.24-3.35) | 0.880 |
| Diuretic | 3.40(1.11-10.38) | 0.032 | 3.02 (1.01-9.07) | 0.048 | 3.39(1.12-10.20) | 0.030 | 4.71(1.39-15.97) | 0.013 |
| Atrial fibrillation | 6.15(1.52-24.95) | 0.011 | 7.37(1.88-28.85) | 0.004 | 5.33(1.34-21.31) | 0.018 | 8.19(1.86-36.08) | 0.005 |
| ECV-MWT (%) | 1.23 (1.02-1.50) | 0.034 | 1.22 (1.01-1.48) | 0.045 | 1.25 (1.03-1.53) | 0.026 | 1.29 (1.05-1.59) | 0.016 |
| LGE (%LV) | 1.02(0.96-1.08) | 0.476 | 1.03(0.97-1.09) | 0.312 | 1.02(0.97-1.08) | 0.450 | 1.02(0.97-1.09) | 0.432 |
| sGLSr (s^–1^) | 13.20(1.50-115.88) | **0.020** |  |  |  |  |  |  |
| sGRSr (s^–1^) |  |  | 4.88(1.20-19.80) | **0.027** |  |  |  |  |
| sGCSr (s^–1^) |  |  |  |  | 4.93(1.05-23.11) | **0.043** |  |  |
| eGLSr (s^–1^) |  |  |  |  |  |  | 24.65(2.89-210.15) | **0.003** |

BMI, Body mass index; ECV, extracellular matrix volume fraction; MWT, maximal wall thickness; LGE, late gadolinium enhancement; sGLSr, sGCSr and sGRSr, global peak systolic LS, CS, RS rate; eGLSr, global early peak diastolic LS rate.

Modle1= sGLSr, Modle2= sGRSr, Modle3= sGCSr, Modle4= eGLSr.

**Supplementary table 2. Interobserver and intraobserver variability of strain measurements**

| Parameters | Interobserver variability | | Intraobserver variability | |
| --- | --- | --- | --- | --- |
|  | ICC | 95%CI | ICC | 95%CI |
| LV-GRS (%) | 0.947 | 0.872-0.979 | 0.968 | 0.921-0.987 |
| LV-sGRSr (s^–1^) | 0.873 | 0.711-0.947 | 0.986 | 0.965-0.994 |
| LV-GCS (%) | 0.889 | 0.743-0.954 | 0.922 | 0.814-0.968 |
| LV-sGCSr (s^–1^) | 0.890 | 0.743-0.955 | 0.905 | 0.766-0.962 |
| LV-GLS (%) | 0.835 | 0.627-0.931 | 0.916 | 0.803-0.966 |
| LV-sGLSr (s^–1^) | 0.870 | 0.702-0.946 | 0.874 | 0.708-0.948 |
| LV-eGLSr (s–1) | 0.810 | 0.579-0.920 | 0.892 | 0.747-0.956 |

ICC, intraclass correlation coefficient; CI, confidence interval; LV, left ventricular; GLS, GCS, and GRS, global peak longitudinal, circumferential, radial strain; sGLSr, sGCSr and sGRSr, global peak systolic LS, CS, RS rate. eGLSr, global early peak diastolic LS rate.

**Figure legends**

**
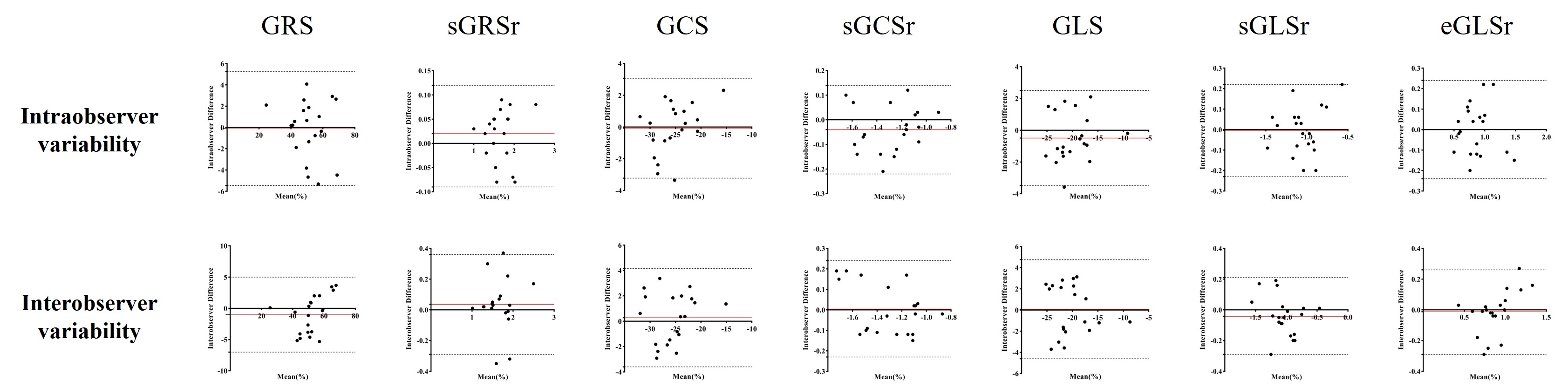
**

**Supplementary Figure 1.** Bland–Altman Plots for intra- and inter-observer variability. Bland–Altman Plots for intra- and inter-observer variability obtained for left ventricular global peak radial (GRS), circumferential (GCS), and longitudinal (GLS) strain, as well as systolic strain rates (sGRSr, sGCSr, sGLSr) and early-diastolic longitudinal strain rate (eGLSr).
